# Prognostic nutritional index as an early predictor of mortality in patients with severe fever with thrombocytopenia syndrome: multicenter retrospective study in South Korea

**DOI:** 10.1186/s12879-025-10661-8

**Published:** 2025-02-25

**Authors:** Hyun Ji Woo, Tae-Kyu Kwon, Sang Taek Heo, Jeong Rae Yoo, Misun Kim, Jaeseong Oh, In-Gyu Bae, Sohyun Bae, Young-Ran Yoon, Miri Hyun, Hyun ah Kim, Sook In Jung, Ki Tae Kwon, Soyoon Hwang, Uh Jin Kim, Gaeun Kang, Young Jun Kim, Jeong-Hwan Hwang, Min-Gul Kim

**Affiliations:** 1 Department of Healthcare Engineering, Graduate School, Jeonbuk National University, Jeonju, Republic of Korea; 2 Nanum Space Co., Ltd, Jeonju, Jeonbuk Republic of Korea; 3 Division of Biomedical Engineering, College of Engineering, Jeonbuk National University, Jeonju, Republic of Korea; 4 Division of Infectious Diseases, Department of Internal Medicine, Jeju National University Hospital, Jeju National University School of Medicine, Jeju, Republic of Korea; 5 Department of Pharmacology, Jeju National University School of Medicine, Jeju National University Hospital, Jeju, Republic of Korea; 6 Division of Infectious Diseases, Department of Internal Medicine, Gyeongsang National University Hospital, Gyeongsang National University School of Medicine, Jinju, Republic of Korea; 7 Division of Infectious Diseases, Department of Internal Medicine, Kyungpook National University Hospital, Kyungpook National University School of Medicine, Daegu, Republic of Korea; 8 Department of Clinical Pharmacology, Kyungpook National University Hospital, Kyungpook National University School of Medicine, Daegu, Republic of Korea; 9 Division of Infectious Diseases, Department of Internal Medicine, Keimyung University Dongsan Hospital, Keimyung University School of Medicine, Daegu, Republic of Korea; 10 Division of Infectious Diseases, Department of Internal Medicine, Chonnam National University Hospital, Chonnam National University Medical School, Gwangju, Republic of Korea; 11 Division of Infectious Diseases, Department of Internal Medicine, Kyungpook National University Chilgok Hospital, Kyungpook National University School of Medicine, Daegu, Republic of Korea; 12 Division of Infectious Diseases, Department of Internal Medicine, Chonnam National University Hwasun Hospital, Chonnam National University School of Medicine, Gwangju, Republic of Korea; 13 Division of Clinical Pharmacology, Chonnam National University Hospital, Gwangju, Republic of Korea; 14 Division of Infectious Diseases, Department of Internal Medicine, Wonkwang University Hospital, Iksan, Republic of Korea; 15 Division of Infectious Diseases, Department of Internal Medicine, Jeonbuk National University Hospital, Jeonbuk National University Medical School, Jeonju, Republic of Korea; 16 Department of Pharmacology, Jeonbuk National University Medical School, Jeonju, Republic of Korea

**Keywords:** Severe fever with thrombocytopenia syndrome, Prognostic nutritional index, Early predictor, Biomarker

## Abstract

**Background and aim:**

Severe fever with thrombocytopenia syndrome (SFTS) is a fatal tick-borne infectious disease lacking effective treatments or vaccines. Early identification of prognostic factors is essential for optimizing clinical management. This study investigated the predictors for mortality in SFTS patients.

**Methods:**

We conducted a retrospective multicenter cohort study of 413 SFTS patients hospitalized in South Korea from 2013 to 2024. Clinical and laboratory data were comprehensively analyzed to evaluate associations between in-hospital mortality and various inflammatory, immune, and nutritional biomarkers. Cox regression and time-dependent receiver operating characteristic (ROC) analyses were performed to identify risk factors.

**Results:**

413 patients diagnosed with SFTS were included and In-hospital mortality was 17% (70/413). Multivariate Cox regression identified older age (HR: 1.042; 95% CI: 1.014–1.071), elevated PT(INR) (HR: 109.57; 95% CI: 19.79–606.57), and lower prognostic nutritional index (PNI) (HR: 0.937; 95% CI: 0.886–0.990) as early predictors of mortality. Time-dependent ROC analysis demonstrated predictive accuracy, with AUCs of 0.512 for age, 0.857 for PT(INR), and 0.694 for PNI at 30 days. Kaplan-Meier analysis revealed significant survival differences for patients stratified by PNI (< 40.75), PT(INR) (≥ 0.97), and age (≥ 59 years).

**Conclusions:**

PNI, PT(INR), and age were identified as key early predictors of mortality in SFTS. PNI, as a novel biomarker, was found to be a useful index for risk level and treatment strategies in SFTS patients.

**Clinical trial number:**

Not applicable.

**Supplementary Information:**

The online version contains supplementary material available at 10.1186/s12879-025-10661-8.

## Introduction

Severe fever with thrombocytopenia syndrome (SFTS) is an emerging tick-borne infectious disease first identified in China in 2009 and subsequently reported in other Asian countries, including South Korea, Japan, and Vietnam [1–4]. In 2014, the pathogen was officially classified as the SFTS virus by the International Committee on Taxonomy of Viruses, and in 2019, it was renamed *Dabie bandavirus* and reclassified into the genus *Bandavirus*, family *Phenuiviridae*, and order *Bunyavirales* [5]. SFTS virus is primarily transmitted through tick bites, although human-to-human transmission via close contact has been reported [6–8]. The Heartland virus, which shares genetic similarities with SFTS, has been identified in the United States, indicating the potential for global dissemination of SFTS. In 2017, the World Health Organization (WHO) classified SFTS as one of the top 10 priority infectious diseases requiring urgent research, alongside Ebola [9].

The common symptoms of SFTS include high fever, fatigue, headache, myalgia, abdominal pain, vomiting, diarrhea, and cough, which are nonspecific. Frequent clinical signs include thrombocytopenia, leukopenia, lymphadenopathy, and gastrointestinal bleeding, with an estimated case fatality rate ranging from 16.2 to 30% [10]. The pathophysiology of SFTS is still poorly understood, and there are currently no effective vaccines or antiviral therapies for prevention or treatment. Most studies have focused on the clinical features and laboratory findings of SFTS, which are insufficient to predict disease severity or guide therapeutic strategies accurately.

SFTS is closely associated with immune dysregulation, which plays a critical role in its pathogenesis, determining the progression and severity of the disease [11–13]. Excessive activation of circulating leukocytes, particularly in response to viremia, reflects systemic inflammation resulting from a failure of cell-intrinsic immunity to control the intracellular virus in leukocytes or other cells. Specifically, SFTS virus triggers an exaggerated inflammatory response — characterized by the excessive production of proinflammatory cytokines, including IL-1β, IL-6, IL-8, TNF-α, IFN-γ and IL-10 — which correlates with viral load and reflects the severity of the inflammatory state [14, 15]. This cytokine storm is related to severe complications, such as multiple organ dysfunction syndrome, shock, and disseminated intravascular coagulation, ultimately causing increased mortality [16, 17]. As a result, patients with SFTS often have severe systemic inflammation, as well as immune dysregulation, hepatic dysfunction, and coagulation abnormalities. Several studies have reported that systemic inflammatory biomarkers — such as C-reactive protein (CRP), neutrophil-to-lymphocyte ratio (NLR), platelet-to-lymphocyte ratio (PLR), monocyte-to-lymphocyte ratio (MLR), C-reactive protein-to-lymphocyte ratio (CLR), C-reactive protein-to-albumin ratio (CAR), BUN-to-Albumin Ratio (BAR), and AST-to-ALT Ratio (AAR) — are predictors for the prognosis of patients with SFTS [18–26]. Other inflammatory biomarkers whose association to the prognosis of patients with SFTS has not yet been established include systemic immune-inflammation index (SII), systemic inflammation response index (SIRI), and aggregate index of systemic inflammation (AISI). These biomarkers may help identify high-risk patients early, allowing for appropriate treatment [27–29].

Nutritional status is also important in predicting the prognosis of SFTS mortality because malnutrition can exacerbate systemic inflammation and impair immune competence. Platelet-to-Albumin Ratio (PAR) and prognostic nutritional index (PNI) are representative biomarkers of nutritional and immune status [30, 31]. These indices indicate the interaction between systemic inflammation, immune competence, and nutritional reserves, making them a comprehensive biomarker of disease progression. For example, PNI, calculated from serum albumin levels and total lymphocyte counts, is independently associated with long-term survival in patients with acute heart failure [32]. Lower PNI values indicate hypoalbuminemia and lymphopenia, conditions often consistent with systemic inflammation and impaired immune function in SFTS. Thus, nutritional status indices may be useful in predicting the prognosis of SFTS. While other markers (e.g., NLR, CLR, CRP, SII, or SIRI) mainly focus on inflammatory states or platelet-related parameters, the PNI was initially designed to evaluate both nutritional and immunological status. This dual perspective may be especially relevant in SFTS, where hepatic dysfunction and immune dysregulation frequently occur together [33, 34]. Despite the increasing importance of PNI in various critical illnesses, its role in SFTS prognosis remains insufficiently studied [35–38]. By comparing PNI to other established or emerging biomarkers, it is important to clarify whether PNI offers prognostic value and can serve as a novel, comprehensive index for early risk classification in SFTS.

In this study, we developed and evaluated a predictive model for in-hospital mortality of SFTS patients using various parameters representing the initial clinical status, including demographic, routine laboratory data, and a comprehensive set of inflammatory, immune, and nutritional indices. Our objective was to determine whether these variables can effectively predict mortality and provide a basis for clinical decision-making, thereby assisting early risk classification and supporting timely therapeutic intervention.

## Methods

### Study design and patient selection

This retrospective, multicenter cohort study was conducted nationwide in South Korea, using nine tertiary referral hospitals with infectious diseases specialists. We included patients with a confirmed diagnosis of SFTS who were hospitalized and treated between May 2013 and October 2024. SFTS virus infection was confirmed by detecting viral RNA in patient serum during the acute phase of illness using real-time polymerase chain reaction (RT-PCR) or Conventional reverse transcription PCR. Conventional reverse transcription PCR was used initially but was replaced by RT-PCR in early 2016; thus, most patients in this study were diagnosed by RT-PCR. We excluded patients who were not admitted to the ward, meaning those evaluated only in the outpatient clinic or those who left the hospital against medical advice, resulting in no available in-hospital follow-up data. We also excluded patients with no laboratory tests performed within 1 day of hospital admission, since we lacked baseline laboratory results on the admission day or the following day. This approach ensured consistent time-point measurements for subsequent analyses.

The study protocol was approved by each hospital’s Institutional Review Board. The list of IRBs and IRB approval numbers is provided in Supplementary Table 1. The requirement for informed consent was waived due to the study’s retrospective nature and the use of de-identified patient data.

### Data collection and variable definition

Demographic, clinical, and laboratory data were collected from the electronic medical records of each hospital. Laboratory parameters were obtained explicitly from tests conducted on the day of hospital admission or the day after admission to capture the most clinically relevant values at the acute phase of SFTS. If data from these time points were unavailable, they were recorded as missing. The laboratory data included key hematological and biochemical measures such as white blood cell count, platelet count, aspartate aminotransferase (AST), and albumin. The primary outcome was in-hospital mortality, defined as death occurring during the hospitalization.

The following indices were calculated based on the available laboratory data:

- Neutrophil-to-Lymphocyte Ratio (NLR) = neutrophil/lymphocyte counts.
- Platelet-to-Lymphocyte ratio (PLR) = platelet/lymphocyte counts.
- Monocyte-to-Lymphocyte ratio (MLR) = monocyte/lymphocyte counts.
- Systemic inflammation index (SII) = platelet counts×neutrophil counts/lymphocyte counts.
- Systemic inflammation response index (SIRI) = neutrophil counts×monocyte counts /lymphocyte counts.
- Aggregate systemic inflammation index (AISI) = neutrophil counts×monocyte counts×platelet counts/lymphocyte counts.
- C-reactive protein-to-Lymphocyte Ratio (CLR) = CRP/lymphocyte counts.
- C-reactive protein-to-albumin ratio (CAR) = CRP/albumin.
- BUN-to-ALB ratio (BAR) = BUN/albumin.
- Platelet-to-albumin ratio (PAR) = platelet counts/albumin.
- Prognostic nutritional index (PNI) = albumin (g/dl)×10 + 5×lymphocyte count (×10³/µL).
- AST/ALT ratio (AAR; De Ritis ratio) = AST/ALT.

These indices were selected based on emerging evidence that they may reflect the inflammatory state, as well as nutritional status and immune competence of various infectious and critical illnesses.

### Statistical analysis

All statistical analyses were performed using R software version 4.4.2 (R Foundation for Statistical Computing, Vienna, Austria). Continuous variables were presented as mean ± standard deviation or median (interquartile range), according to data distribution. Categorical variables were expressed as frequencies and percentages. Differences between survivors and non-survivors were evaluated using the Student’s t-test or Mann-Whitney U test for continuous variables and the Chi-square test for categorical variables.

Time-to-event analysis was performed using the Cox proportional hazards regression model with *survival* package. Time-to-event was defined from symptom onset to death (event = 1) or censored at discharge (event = 0). Univariate Cox regression analyses were performed to identify candidate predictors of mortality. Then, variables with *p* < 0.05 were considered in the multivariate Cox model. Least absolute shrinkage and selection operator (LASSO) Cox regression analysis was performed to screen potential prognostic factors using the *glmnet* package. Ten-fold cross-validation was used to select the optimal lambda, with the lambda value (λ_min) that minimizes the cross-validated error subsequently used to fit the LASSO Cox model and extract coefficients corresponding to non-zero predictors. The variables selected by the LASSO Cox regression analysis were then subjected to multivariate Cox regression analysis using a forward selection method to identify independent risk factors for fatal outcomes in SFTS patients. Adjusted hazard ratios (HRs) with 95% confidence intervals (CIs) were reported to identify independent predictors of mortality. Additionally, stratified Cox regression analyses were conducted to assess the prognostic impact of selected predictors across different subgroups defined by age, gender, and hospital location. For the analysis of the predictive model, the pairwise deletion method was used to handle missing data [39]. This method allows the inclusion of available data for each variable individually rather than excluding an entire case when one variable is missing.

To evaluate the predictive performance of the selected variables at specific time points while properly accounting for patients who were censored, we performed a time-dependent receiver operating characteristic (ROC) analysis using the *timeROC* package. Time-dependent ROC curves and corresponding area under the curve (AUC) values were calculated at t = 7 and 30 days for each predictor variable. The AUC was calculated to measure predictive accuracy, and optimal cut-off values were determined using the Youden index to maximize sensitivity and specificity. The key predictor variables were dichotomized based on their respective cut-off value. Kaplan-Meier survival curves were generated for each dichotomized variable, and survival differences were compared using the log-rank test.

To verify the proportional hazards assumption for the Cox regression and Kaplan-Meier analyses, both statistical tests and graphical methods were applied. Specifically, the Schoenfeld residuals test was used to assess the proportional hazards assumption statistically, and log-log survival plots and Schoenfeld residual plots were generated to inspect the assumption visually. No violations of the proportional hazards assumption were detected for the main predictors.

## Results

### Patients characteristics

**Fig. 1:**
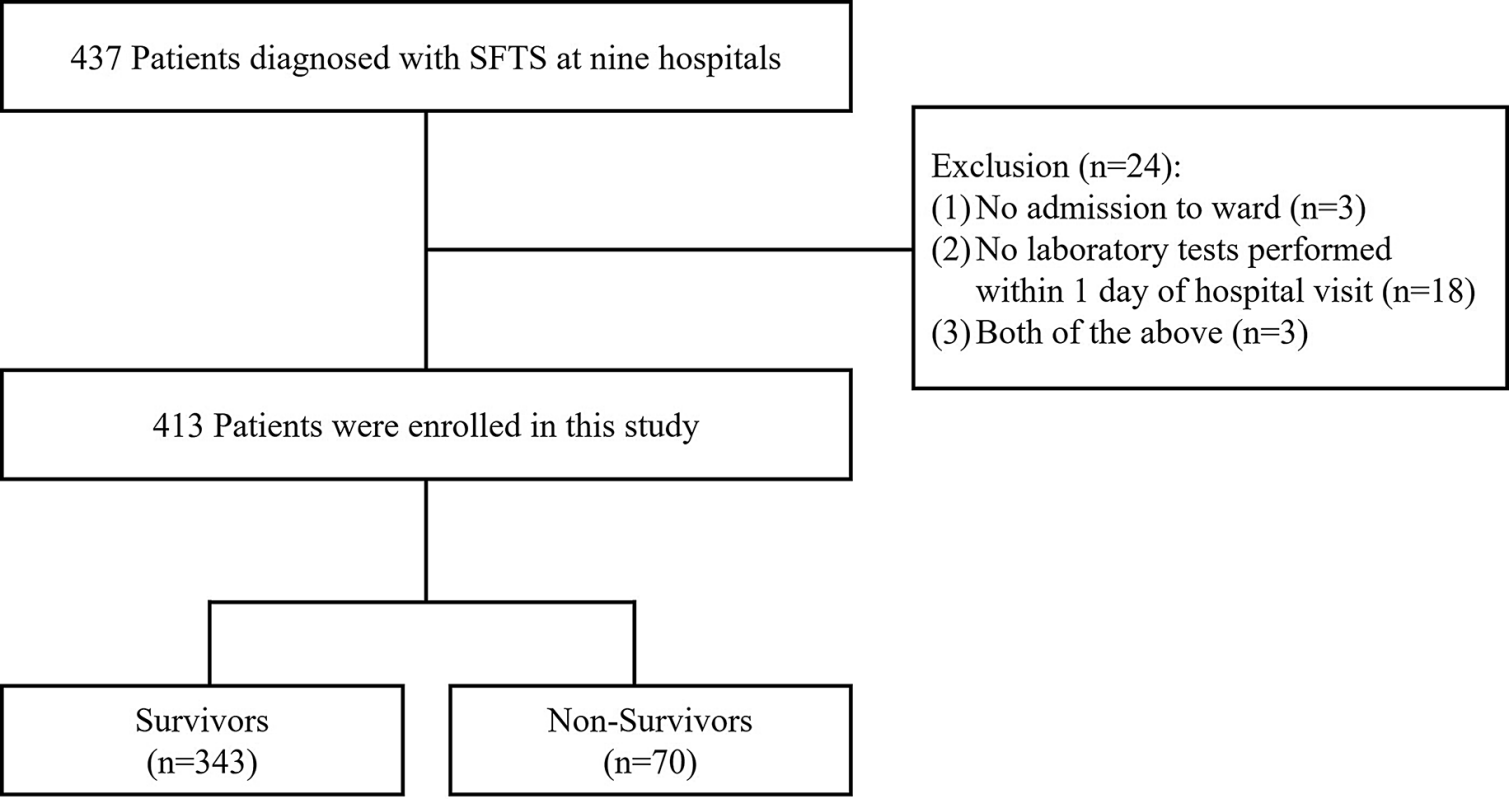
Study Flow Chart of Patient Selection. ‘No admission to ward’ indicates patients were seen only in the outpatient clinic or left the hospital against medical advice, providing no in-hospital follow-up data. ‘No laboratory tests within 1 day’ indicates who had no baseline lab results obtained on the day of admission or the following day

A total of 413 patients diagnosed with SFTS were included in this study (Fig. 1). Baseline demographic and clinical characteristics are presented in Table 1. The non-survivors were older, had a higher prevalence of diabetes mellitus, and had shorter durations from onset of illness to discharge or death (all *p* < 0.05).

**Table 1:** Baseline characteristics of patients with SFTS

Characteristics	Overall (*n* = 413)	Survivors (*n* = 343)	Non-survivor (*n* = 70)	*p*-value
*Demographics*				
Age (year)	67.12 (13.28)	65.84 (13.63)	73.40 (9.20)	< 0.001
Gender, n (%)				
Male	194 (47.0)	163 (47.5)	31 (44.3)	0.717
Female	219 (53.0)	180 (52.5)	39 (55.7)	
Height (cm)	161.79 (9.73)	161.71 (9.99)	162.18 (8.38)	0.735
Weight (kg)	61.34 (13.35)	61.22 (13.33)	61.91 (13.54)	0.705
*Signs & Symptoms*				
Body temperature	37.48 (0.88)	37.48 (0.88)	37.51 (0.87)	0.781
Systolic blood pressure	119.70 (19.60)	119.97 (19.70)	118.39 (19.20)	0.538
Diastolic blood pressure	71.63 (13.69)	71.69 (13.66)	71.34 (13.95)	0.846
Heart rate	81.20 (15.59)	81.00 (15.24)	82.17 (17.28)	0.568
Respiratory rate	19.76 (2.11)	19.69 (2.02)	20.10 (2.47)	0.136
Glasgow coma scale	14.64 (1.32)	14.82 (0.75)	13.76 (2.58)	< 0.001
APACHE II Score	25.51 (4.11)	25.40 (4.03)	26.07 (4.44)	0.211
O_2_ supply, n (%)	70 (16.9)	38 (11.1)	32 (45.7)	< 0.001
Fever, n (%)	298 (72.2)	248 (72.3)	50 (71.4)	0.998
Headache, n (%)	70 (16.9)	57 (16.6)	13 (18.6)	0.824
Dyspnea, n (%)	14 (3.4)	9 (2.6)	5 (7.1)	0.123
*Comorbidities*				
Diabetes, n (%)	70 (16.9)	51 (14.9)	19 (27.1)	0.020
Hypertension, n (%)	154 (37.3)	121 (35.3)	33 (47.1)	0.083
Chronic lung disease, n (%)	47 (11.4)	35 (10.2)	12 (17.1)	0.144
Chronic kidney disease, n (%)	2 (0.5)	1 (0.3)	1 (1.4)	0.311
Chronic liver disease, n (%)	8 (1.9)	7 (2.0)	1 (1.4)	> 0.999
*Duration*				
Onset of illness to admission (days)	4.67 (2.62)	4.71 (2.64)	4.50 (2.52)	0.544
Onset of illness to discharge or death (days)	15.01 (8.99)	16.06 (9.31)	9.87 (4.47)	< 0.001
*Laboratory finding*				
WBC count (×10³/µL)	2.38 (2.01)	2.36 (1.91)	2.49 (2.47)	0.623
Platelet count (×10³/µL)	73.86 (39.88)	76.43 (40.52)	60.83 (33.81)	0.004
Neutrophil count (×10³/µL)	1.45 (1.52)	1.42 (1.44)	1.61 (1.85)	0.360
Lymphocyte count (×10³/µL)	0.70 (0.60)	0.71 (0.61)	0.64 (0.58)	0.404
Monocyte count (×10³/µL)	0.16 (0.18)	0.17 (0.18)	0.12 (0.14)	0.038
Hemoglobin (g/dL)	13.51 (1.93)	13.53 (1.94)	13.46 (1.85)	0.794
Hematocrit (%)	39.89 (5.54)	39.98 (5.61)	39.44 (5.20)	0.463
Sodium (mmol/L)	135.42 (4.40)	135.36 (4.27)	135.73 (5.02)	0.528
Potassium (mmol/L)	3.94 (0.63)	3.93 (0.62)	4.00 (0.66)	0.414
AST (IU/L)	271.12 (373.09)	213.68 (250.16)	557.46 (654.14)	< 0.001
ALT (IU/L)	107.09 (125.35)	97.60 (115.39)	154.14 (158.96)	0.001
LDH (IU/L)	955.63 (1031.54)	785.73 (637.24)	1846.88 (1903.13)	< 0.001
Total Bilirubin (mg/dL)	0.46 (0.38)	0.44 (0.34)	0.56 (0.53)	0.016
Total Protein (g/dL)	6.14 (0.72)	6.21 (0.68)	5.77 (0.80)	< 0.001
Albumin (g/dL)	3.58 (0.52)	3.63 (0.48)	3.28 (0.59)	< 0.001
BUN (mg/dL)	19.60 (13.20)	19.76 (14.15)	18.75 (7.08)	0.845
Creatinine (mg/dL)	1.06 (0.63)	1.03 (0.61)	1.26 (0.70)	0.011
CRP (mg/L)	5.16 (18.10)	4.53 (18.49)	8.70 (15.44)	0.100
Troponin I (ng/mL)	0.77 (4.44)	0.81 (4.94)	0.64 (1.37)	0.860
CK-MB (ng/mL)	8.39 (13.27)	7.32 (12.80)	12.41 (14.33)	0.013
PT (INR)	1.06 (0.14)	1.04 (0.09)	1.16 (0.25)	< 0.001
aPTT (sec)	44.46 (14.82)	42.16 (10.71)	56.16 (24.41)	< 0.001
eGFR (mL/min/1.73 m²)	67.59 (44.13)	66.84 (43.11)	71.95 (49.88)	0.445
*Index based on laboratory data*				
NLR	2.58 (2.41)	2.57 (2.52)	2.65 (1.72)	0.811
PLR	151.99 (106.17)	154.53 (105.89)	138.56 (107.52)	0.274
MLR	0.24 (0.18)	0.25 (0.19)	0.19 (0.14)	0.015
SII	197.28 (223.55)	204.72 (237.09)	157.84 (124.10)	0.127
SIRI	0.44 (0.87)	0.46 (0.91)	0.33 (0.63)	0.257
AISI	38.94 (95.88)	41.85 (101.98)	23.56 (50.70)	0.165
CLR	9.77 (33.51)	8.47 (33.30)	17.15 (34.07)	0.066
CAR	1.58 (5.77)	1.33 (5.58)	2.99 (6.63)	0.040
BAR	5.98 (4.37)	5.90 (4.61)	6.39 (3.15)	0.777
PAR	20.30 (9.47)	20.61 (9.47)	18.70 (9.36)	0.141
PNI	39.29 (5.53)	39.89 (5.40)	36.23 (5.19)	< 0.001
AAR	2.47 (1.26)	2.30 (1.10)	3.35 (1.61)	< 0.001

Data are presented as mean (standard deviation) for continuous variables and count (percentage) for categorical variables. Laboratory findings and variable index are evaluated among available data

*Abbreviations* SFTS, Severe fever with thrombocytopenia syndrome; APACHE II Score, Acute Physiology and Chronic Health Evaluation II Score; AST, Aspartate aminotransferase; ALT, alanine aminotransferase; LDH, lactate dehydrogenase; BUN, blood urea nitrogen; CRP, C-reactive protein; CK-MB, creatine phosphokinase MB fraction; PT(INR), prothrombin time (International Normalized Ratio); aPTT, activated partial thromboplastin time; eGFR, estimated glomerular filtration rate; NLR, Neutrophil-to-Lymphocyte Ratio; PLR, Platelet-to-Lymphocyte Ratio; MLR, Monocyte-to-Lymphocyte Ratio; SII, Systemic Immune-Inflammation Index; SIRI, Systemic Inflammatory Response Index; AISI, Aggregate Index of Systemic Inflammation; CLR, CRP-to-Lymphocyte Ratio; CAR, CRP-to-Albumin Ratio; BAR, BUN-to-Albumin Ratio; PAR, Platelet-to-Albumin Ratio; PNI, Prognostic Nutritional Index; AAR, AST-to-ALT Ratio

Laboratory findings at admission also differed between the two groups (Table 1). Significant differences in laboratory parameters (platelet count, albumin, AST, ALT, PT[INR], etc.) indicate hepatic dysfunction, coagulopathy, and elevated inflammatory state in non-survivors. In particular, the mean PNI was lower in non-survivors (36.23 vs. 39.89, *p* < 0.001).

### Independent risk factors for mortality in patients with SFTS

In univariate Cox proportional hazards regression analyses (Table 2), several variables were associated with an increased hazard of in-hospital mortality. Age, various laboratory biomarkers (e.g., platelet count, AST, ALT, LDH, total bilirubin, total protein, albumin, creatinine, PT(INR), and aPTT) showed statistically significant associations. Among the calculated indices, higher AAR and lower MLR and PNI were each significantly correlated with an increased risk of death (all *p* < 0.05). LASSO Cox regression analysis identified age, AST, creatinine, PT(INR), aPTT, LDH, PNI, and AAR as candidate predictors, which were subsequently subjected to a multivariate Cox regression model. In the final multivariate Cox regression model, older Age (HR 1.042, 95% CI 1.014–1.071, *p* = 0.003), elevated PT(INR) (HR 109.565, 95% CI 19.791–606.569, *p* < 0.001), and lower PNI (HR 0.937, 95% CI 0.886–0.990, *p* = 0.022) remained independently associated with increased mortality risk. Detailed results of the univariate and multivariate Cox regression are shown in Table 2.

**Table 2:** Risk factors associated with disease prognosis of patients with SFTS

Variable	Univariate cox regression		Multivariate cox regression	
	Hazards ratio (95% CI)	*p*-value	Hazards ratio (95% CI)	*p*-value
Age	1.040 (1.017, 1.063)	0.001	1.042 (1.014, 1.071)	0.003
Gender	1.068 (0.666, 1.712)	0.786		
Height	1.006 (0.980, 1.032)	0.645		
Weight	1.008 (0.990, 1.026)	0.396		
WBC count	1.017 (0.917, 1.127)	0.749		
Neutrophil count	1.045 (0.918, 1.190)	0.506		
Lymphocyte count	0.860 (0.534, 1.385)	0.536		
Monocyte count	0.148 (0.022, 1.010)	0.051		
Hemoglobin	1.000 (0.889, 1.125)	0.998		
Hematocrit	0.991 (0.951, 1.032)	0.656		
Platelet count	0.990 (0.982, 0.998)	0.018		
Sodium	1.002 (0.969, 1.073)	0.450		
Potassium	1.144 (0.855, 1.530)	0.364		
AST	1.001 (1.001, 1.001)	< 0.001		
ALT	1.002 (1.001, 1.003)	0.001		
LDH	1.000 (1.000, 1.001)	< 0.001		
Total Bilirubin	1.550 (1.009, 2.381)	0.045		
Total Protein	0.512 (0.364, 0.720)	< 0.001		
Albumin	0.394 (0.257, 0.604)	< 0.001		
BUN	0.990 (0.927, 1.057)	0.754		
Creatinine	1.449 (1.073, 1.956)	0.016		
CRP	1.006 (0.997, 1.014)	0.176		
Troponin I	0.981 (0.885, 1.087)	0.710		
CK-MB	1.013 (1.000, 1.027)	0.056		
PT(INR)	12.304 (5.969, 25.362)	< 0.001	109.565 (19.791, 606.569)	< 0.001
aPTT	1.028 (1.020, 1.037)	< 0.001		
eGFR	1.002 (0.997, 1.008)	0.414		
NLR	1.022 (0.916, 1.140)	0.700		
PLR	0.999 (0.996, 1.001)	0.332		
MLR	0.109 (0.017, 0.694)	0.019		
SII	0.999 (0.997, 1.001)	0.200		
SIRI	0.774 (0.497, 1.207)	0.258		
AISI	0.996 (0.990, 1.002)	0.192		
CLR	1.004 (1.000, 1.008)	0.052		
CAR	1.020 (0.995, 1.045)	0.114		
BAR	1.018 (0.865, 1.198)	0.829		
PAR	0.983 (0.955, 1.012)	0.257		
PNI	0.903 (0.861, 0.948)	< 0.001	0.937 (0.886, 0.990)	0.022
AAR	1.324 (1.183, 1.481)	< 0.001		

*Abbreviations* SFTS, Severe fever with thrombocytopenia syndrome; CI, confidence interval; AST, Aspartate aminotransferase; ALT, alanine aminotransferase; LDH, lactate dehydrogenase; BUN, blood urea nitrogen; CRP, C-reactive protein; CK-MB, creatine phosphokinase MB fraction; PT(INR), prothrombin time (International Normalized Ratio); aPTT, activated partial thromboplastin time; eGFR, estimated glomerular filtration rate; NLR, Neutrophil-to-Lymphocyte Ratio; PLR, Platelet-to-Lymphocyte Ratio; MLR, Monocyte-to-Lymphocyte Ratio; SII, Systemic Immune-Inflammation Index; SIRI, Systemic Inflammatory Response Index; AISI, Aggregate Index of Systemic Inflammation; CLR, CRP-to-Lymphocyte Ratio; CAR, CRP-to-Albumin Ratio; BAR, BUN-to-Albumin Ratio; PAR, Platelet-to-Albumin Ratio; PNI, Prognostic Nutritional Index; AAR, AST-to-ALT Ratio

Stratified Cox regression analyses were performed across subgroups defined by age (< 65 vs. ≥65 years), gender (male vs. female), and hospital location (eastern vs. western region of South Korea). PNI was significantly associated with mortality risk in all subgroups except females. (see Supplementary Table 2 for detailed results)

### Time-dependent ROC analysis and predictive performance

**Fig. 2:**
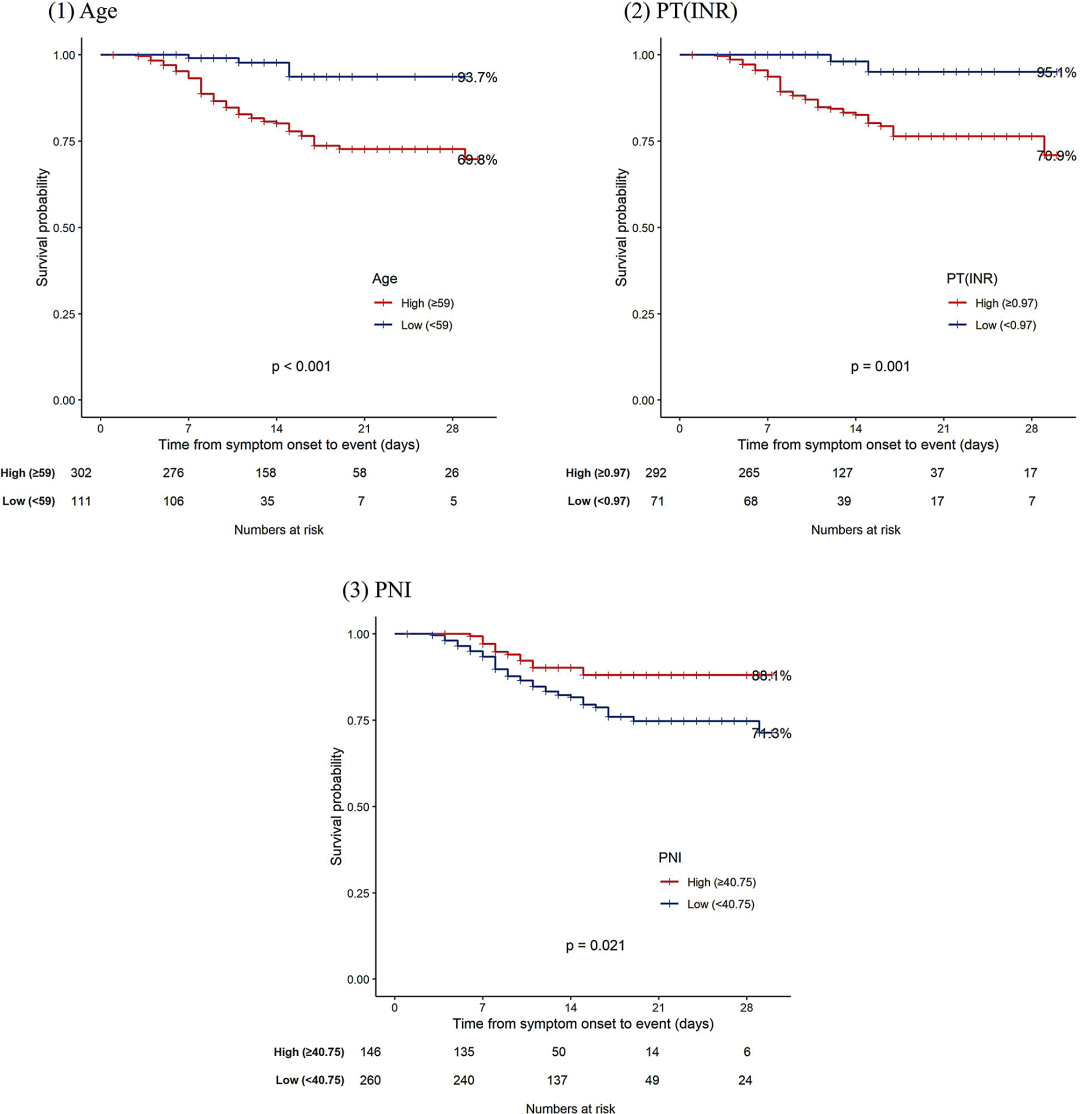
Kaplan-Meier Survival curves stratified by key prognostic biomarkers in patients with SFTS. *Abbreviations* SFTS, Severe fever with thrombocytopenia syndrome; PT(INR), prothrombin time (International Normalized Ratio); PNI, Prognostic Nutritional Index

The time-dependent ROC analysis results for age, PT(INR), and PNI are summarized in Table 3. At t = 7 days, age showed an AUC of 0.648 with a cut-off of 59, PT(INR) demonstrated an AUC of 0.692 with a cut-off of 1.01, and PNI presented an AUC of 0.716 with a cut-off of 38.78. At t = 30 days, age showed an AUC of 0.512 with a cut-off of 59, PT(INR) demonstrated an AUC of 0.857 with a cut-off of 0.97, and PNI presented an AUC of 0.694 with a cut-off of 40.75. Kaplan-Meier survival curves stratified by PNI, PT(INR), and age showed significant survival probability. Patients with low PNI (< 40.75), elevated PT(INR) (≥ 0.97), or older Age (≥ 59 years) had significantly reduced survival probabilities (log-rank test, *p* < 0.05 for all comparisons) (Fig. 2). These findings suggest that these predictors may be helpful in identifying patients at high risk of mortality.

**Table 3:** Time-dependent predictive performance of selected variables in multivariable Cox regression for SFTS mortality

Variable	Time (day)	AUC	Cut-off value	Sensitivity	Specificity	*p*-value
Age	7	0.648	59	> 0.999	0.279	< 0.001
Age	30	0.512	59	0.943	0.274	0.545
PT(INR)	7	0.692	1.01	0.923	0.366	< 0.001
PT(INR)	30	0.857	0.97	0.956	0.593	< 0.001
PNI	7	0.716	38.78	0.856	0.540	< 0.001
PNI	30	0.694	40.75	0.797	0.632	< 0.001

*Note* The cutoff points were selected by maximizing the sum of sensitivity and specificity

*Abbreviations* SFTS, Severe fever with thrombocytopenia syndrome; AUC, area under the curve; CI, confidence interval; PT(INR), Prothrombin Time International Normalized Ratio; PNI, Prognostic Nutritional Index

## Discussion

SFTS is a disease that causes a significant public health challenge characterized by high mortality and rapid progression, especially in vulnerable populations such as the elderly and immunocompromised [2–4, 40, 41]. Reliable early predictors of disease prognosis are essential to optimizing clinical decision-making and improving patient outcomes. In this study, we defined early prognostic factors as biomarkers or clinical indicators that can be identified at the time of hospital admission or within the first 24 h of admission. Late prognostic factors, on the other hand, are those that become apparent or significant after the initial 24-hour period, often representing the progression of the disease and the patient’s response to the initial treatments. Our study, based on a cohort of 413 patients from 9 tertiary referral hospitals in South Korea, found three early predictors, age, PT(INR), and PNI, in providing unique insights into the pathophysiology and clinical progression of SFTS. That is, older age, high PT(INR), and low PNI are independent predictors of mortality in patients with SFTS. Moreover, ROC analysis showed that PT (INR), PNI, and age have good predictive power for in-hospital mortality in patients with SFTS, with PT(INR) being the most predictive.

In this study, indices such as NLR, PLR, MLR, SII, SIRI, AISI, CLR, CAR, BAR, PAR, and AAR, did not show any predictive power for the prognosis of patients with SFTS. This may be due to the specific immunopathogenesis of SFTS, in which other immune and coagulation factors, such as age and PT(INR), are more prominent. MLR and AAR showed some potential for predicting mortality in univariate Cox analyses, but they were not statistically significant in multivariate analyses. Nevertheless, these markers might still be useful in resource-limited settings.

Patients with SFTS often experience hepatic dysfunction, which further worsens inflammation and poor nutritional status [42]. The PNI is considered a novel biomarker of the nutritional and immune status [43, 44]. It is calculated using serum albumin levels and total lymphocyte counts and provides a comprehensive assessment of a patient’s nutritional and immune status. In this study, lower PNI was associated with increased mortality, which indicates the importance of PNI as an independent prognostic biomarker in SFTS. Hypoalbuminemia, a major component of PNI, reflects poor nutrient reserves and systemic inflammation [45]. Additionally, lymphopenia, another important component of PNI, suggests that immune competence is impaired, making patients more susceptible to secondary infections and reducing their ability to control their viral load. As a readily available and cost-effective biomarker, PNI can help identify high-risk groups that may benefit from targeted nutrition and immune-boosting interventions. For example, improving albumin levels through diet or supplement support and addressing lymphocyte deficiency with immunomodulatory therapy may mitigate the risks associated with low PNI.

Although our findings demonstrate that low PNI is significantly associated with higher mortality, the current data do not sufficiently support that low PNI alone necessitates early intervention such as close monitoring and more active supportive care. Rather, PNI may improve previously reported prognostic models by considering both nutritional and immune status, thereby assisting in risk classification and potentially informing timely supportive care. However, further prospective studies are needed to determine whether targeted interventions based on low PNI effectively improve mortality.

In this study, age and PT(INR) were also found to be significant predictors of mortality. Consistent with previous studies, older age is associated with worse outcomes in infectious diseases, mainly due to immunosenescence and comorbidities [46]. Prolonged PT(INR) indicates coagulopathy and liver dysfunction, which play an important role in the pathophysiology of SFTS [47]. Unlike other biomarkers that may focus on a single pathophysiological mechanism, PNI represents multiple physiological domains (nutritional status and immune competence), offering a more comprehensive perspective compared to other established predictors such as age and PT(INR).

### Limitations

Due to the retrospective design of the study, it may cause biases related to data collection and interpretation. First, missing data was inevitable, and our use of the pairwise deletion method to handle it may have led to the potential for selection bias. Second, the study did not include viral load measurements, which could have provided additional information on disease progression and prognostic implications, because viral load measurements are not conducted in routine clinical practice. Third, since only in-hospital mortality was recorded, any deaths occurring after discharge were not collected, potentially underestimating the overall mortality associated with SFTS. The limitation of using LASSO regression for variable selection is that the shrinkage penalty may exclude predictors with borderline significance, especially when multicollinearity is present. Although 10-fold cross-validation was used to select the optimal lambda (λ), this approach may bias effect size estimates.

## Conclusions

In this nationwide multicenter study of 413 patients with SFTS, we identified older age, elevated PT(INR), and low PNI as early predictors of in-hospital mortality in SFTS patients. In particular, PNI is a novel early predictor that has not been previously studied. Our findings suggest that enhanced prognostic models with PNI may improve the precision of mortality prediction. However, the relatively small sample size may constrain the robustness of the identified predictors. Therefore, further studies with larger, prospectively designed, multicenter cohorts are needed to validate these results.

## Electronic supplementary material

Below is the link to the electronic supplementary material.

Supplementary Material 1

## Data Availability

The datasets generated and/or analysed during the current study are not publicly available due to secrecy.
