# Supplementary material for: Prognostic nutritional index as an early predictor of mortality in patients with severe fever with thrombocytopenia syndrome: multicenter retrospective study in South Korea
Source: BMC Infect Dis. 2025 Feb 25;25:274. doi: 10.1186/s12879-025-10661-8 (PMC11863440; doi:10.1186/s12879-025-10661-8)
Supplement: Supplementary file 1 — Supplementary Material 1 [file 12879_2025_10661_MOESM1_ESM.docx]

Supplementary Table 1. List of IRBs and IRB approval numbers

| Name of IRBs | Contact number | IRB approval number |
| --- | --- | --- |
| Chonnam National University Hospital | +82-62-220-5257 | CNUH-2023-213 |
| Chonnam National University Hwasun Hospital | +82-61-379-7598 | CNUH-2024-150 |
| Gyeongsang National University Hospital | +82-55-750-9252 | GNUH 2024-08-020 |
| Jeju National University Hospital | +82-64-717-1503 | JEJUNUH 2024-08-002 |
| Jeonbuk National University Hospital | +82-63-259-3339 | CUH 2022-01-067 |
| Keimyung University Dongsan Hospital | +82-53-258-6693 | DSMC 2024-08-008 |
| Kyungpook National University Chilgok Hospital | +82-53-200-2162 | DGIRB 2022-05-004 |
| Kyungpook National University Hospital | +82-53-200-5430 | DGIRB 2023-07-001 |
| Wonkwang University Hospital | +82-63-859-2234 | WKUH 2024-08-005 |

IRB, Institutional Review Board

Supplementary Table 2. Results of stratified Cox regression analysis for age, PT(INR), and prognostic nutritional index (PNI)

| Variable | Subgroup | No. of Patients | No. of Events | Event Rate (%) | Hazards Ratio (95% CI) | p-value |
| --- | --- | --- | --- | --- | --- | --- |
| Age | *Gender* |  |  |  |  |  |
|  | Male | 176 | 26 | 15.5 | 1.037 (1.000, 1.076) | 0.053 |
|  | Female | 183 | 27 | 16.9 | 1.057 (1.013, 1.101) | 0.010 |
|  | *Hospital Location in South Korea* | | |  |  |  |
|  | Eastern Region | 185 | 31 | 16.8 | 1.041 (1.007, 1.076) | 0.018 |
|  | Western Region | 174 | 22 | 12.6 | 1.036 (0.997, 1.075) | 0.070 |
| PT(INR) | *Age* |  |  |  |  |  |
|  | ≥60 years | 202 | 42 | 20.8 | 164.749 (28.628, 948.119) | <0.001 |
|  | <60 years | 157 | 11 | 7.0 | 273.732 (6.886, 10881.057) | 0.003 |
|  | *Gender* |  |  |  |  |  |
|  | Male | 176 | 26 | 15.5 | 110.996 (12.350, 997.586) | <0.001 |
|  | Female | 183 | 27 | 16.9 | 438.753 (36.493, 5275.093) | <0.001 |
|  | *Hospital Location in South Korea* | | |  |  |  |
|  | Eastern Region | 185 | 31 | 16.8 | 77.391 (9.390, 637.874) | <0.001 |
|  | Western Region | 174 | 22 | 12.6 | 651.02 (54.295, 7806.071) | <0.001 |
| PNI | *Age* |  |  |  |  |  |
|  | ≥60 years | 202 | 42 | 20.8 | 0.920 (0.861, 0.983) | 0.014 |
|  | <60 years | 157 | 11 | 7.0 | 0.837 (0.754, 0.930) | 0.001 |
|  | *Gender* |  |  |  |  |  |
|  | Male | 193 | 30 | 15.5 | 0.845 (0.793, 0.900) | <0.001 |
|  | Female | 213 | 36 | 16.9 | 0.971 (0.907, 1.038) | 0.385 |
|  | *Hospital Location in South Korea* | | |  |  |  |
|  | Eastern Region | 185 | 31 | 16.8 | 0.898 (0.838, 0.962) | 0.002 |
|  | Western Region | 174 | 22 | 12.6 | 0.863 (0.794, 0.937) | <0.001 |

Abbreviations: CI, confidence interval; No, number; PT(INR), prothrombin time (International Normalized Ratio); PNI, prognostic nutritional index
